# Chondroitinase ABC Treatment Improves the Organization and Mechanics of 3D Bioprinted Meniscal Tissue

**DOI:** 10.1021/acsbiomaterials.3c00101

**Published:** 2023-05-16

**Authors:** Xavier Barceló, Orquidea Garcia, Daniel J. Kelly

**Affiliations:** † Trinity Centre for Biomedical Engineering, Trinity Biomedical Sciences Institute, Trinity College Dublin, Dublin D02 R590, Ireland; ‡ Department of Mechanical, Manufacturing, & Biomedical Engineering, School of Engineering, Trinity College Dublin, Dublin D02 R590, Ireland; § Advanced Materials & Bioengineering Research Centre (AMBER), Royal College of Surgeons in Ireland & Trinity College Dublin, Dublin D02 F6N2, Ireland; ∥ Johnson & Johnson 3D Printing Innovation & Customer Solutions, Johnson & Johnson Services, Inc., Dublin D02 R590, Ireland; ⊥ Department of Anatomy and Regenerative Medicine, Royal College of Surgeons in Ireland, Dublin D02 YN77, Ireland

**Keywords:** melt electrowriting (MEW), meniscus, bioprinting, collagen, chondroitinase ABC

## Abstract

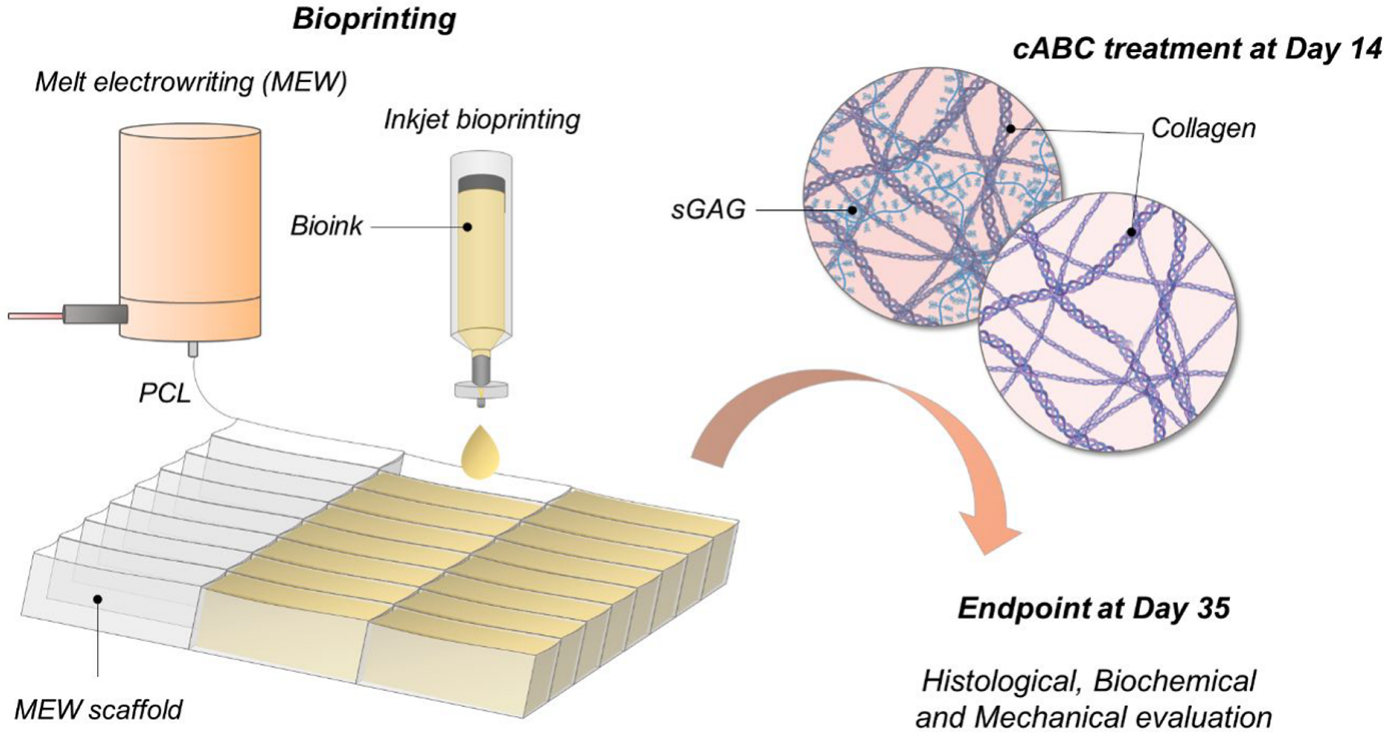

The meniscus is a fibrocartilage tissue that is integral to the correct functioning of the knee joint. The tissue possesses a unique collagen fiber architecture that is integral to its biomechanical functionality. In particular, a network of circumferentially aligned collagen fibers function to bear the high tensile forces generated in the tissue during normal daily activities. The limited regenerative capacity of the meniscus has motivated increased interest in meniscus tissue engineering; however, the *in vitro* generation of structurally organized meniscal grafts with a collagen architecture mimetic of the native meniscus remains a significant challenge. Here we used melt electrowriting (MEW) to produce scaffolds with defined pore architectures to impose physical boundaries upon cell growth and extracellular matrix production. This enabled the bioprinting of anisotropic tissues with collagen fibers preferentially oriented parallel to the long axis of the scaffold pores. Furthermore, temporally removing glycosaminoglycans (sGAGs) during the early stages of *in vitro* tissue development using chondroitinase ABC (cABC) was found to positively impact collagen network maturation. Specially we found that temporal depletion of sGAGs is associated with an increase in collagen fiber diameter without any detrimental effect on the development of a meniscal tissue phenotype or subsequent extracellular matrix production. Moreover, temporal cABC treatment supported the development of engineered tissues with superior tensile mechanical properties compared to empty MEW scaffolds. These findings demonstrate the benefit of temporal enzymatic treatments when engineering structurally anisotropic tissues using emerging biofabrication technologies such as MEW and inkjet bioprinting.

## 1 Introduction

The meniscus is a crescent-shaped fibrocartilage tissue that is integral to the functionality of the knee joint, acting to maintain stability and distributing loads across the joint surface.^1−3^ The mechanical function of the meniscus is tightly linked to the unique architecture of the collagen fibers within the tissue, which are preferentially oriented in the circumferential direction, enabling the tissue to bear high levels of load.^4^ Injuries to the meniscus disrupt the normal functioning of the knee joint and can eventually can lead to the development of osteoarthritis.^5^ Current clinical treatments for damaged menisci, such as partial or total meniscectomy, only provide short-term relief.^6^ This has led to increased interest in the development of biomaterial implants to provide structural support and serve as a template for new tissue formation.^7,8^ However, none of the clinically available biomaterial substitutes closely recapitulate the intricate organization of the native meniscus that is key to its correct functionality. In this context, 3D bioprinting is emerging as a biofabrication technique with the ability to produce regenerative constructs that mimic the composition and key architectural features of living tissues.^9^

Different 3D bioprinting strategies have already been employed to produce biomimetic constructs for meniscus tissue engineering.^10,11^ A common approach has been to use synthetic polymers to fabricate porous scaffolds with bulk mechanical properties comparable to the native tissue.^12−15^ Such scaffolds can be further functionalized with factors supportive of tissue regeneration; for example, the inclusion of growth factor-containing microspheres at specific locations within polycaprolactone (PCL)-based scaffolds has been shown to support the development of zone-specific cellular phenotypes mimetic of the native meniscus.^16^ It has also been demonstrated that the application of both biochemical and biomechanical stimuli to cell-seeded PCL scaffolds can synergistically enhance meniscus zonal organization *in vitro* and *in vivo*.^17^ Cellular constructs have been then fabricated by sequentially printing cell-laden bioinks within supporting PCL scaffolds.^18,19^ The majority of such 3D printed grafts aim to replicate certain aspects of the native tissue anisotropy through the circumferential patterning of polymeric materials like PCL. Although this enables the production of scaffolds with promising bulk mechanical properties, other features of the tissue (e.g., tension-compression nonlinearity) are difficult to replicate, and the key challenge of engineering grafts with a collagen architecture mimetic of the native meniscus has yet to be adequately addressed.^20^

The structural guidance of cells by physical cues is a powerful approach to direct tissue formation and maturation.^21,22^ Diverse strategies, from the use of microgrooves to the electrospinning of aligned fibrous polymers, haven been explored as physical guiding templates to control cellular orientation and/or collagen organization.^23−26^ However, such approaches are generally not suitable for the biofabrication of large anatomically defined implants, which is particularly important for functional meniscus regeneration, thereby motivating the use of emerging 3D bioprinting strategies to address this challenge.^27^ We have previously reported a convergent bioprinting strategy that enables the engineering of soft tissues with user defined collagen network architectures and anisotropic mechanical properties.^28−30^ Using melt electrowriting (MEW) to produce scaffolds with precise, predefined architectures, we were able to alter the physical boundaries imposed upon cells and their secreted extracellular matrix. In MEW scaffolds with highly elongated pore architectures, the deposited ECM preferentially aligned parallel to the long axis of the printed pores, enabling the bioprinting of tissues with collagen organization somewhat mimetic of the native meniscus. Although the compressive properties of the resulting tissues were within the range of native values (90–160 kPa),^31^ the tensile properties, which are integral to tissue functionality, were still orders of magnitude below native values. It has previously been shown that the application of enzymes like chondroitinase ABC (cABC), which temporally remove sulfated glycosaminoglycansing (sGAGs) from the developing tissue, can enhance collagen fiber maturation and hence increase the mechanical properties of engineered cartilage and meniscus.^20,32−37^ In the context of articular cartilage tissue engineering, it has also been shown that such treatments can support the formation of a more zonally organized collagen architecture.^38^ Therefore, the goal of this study was to determine the impact of such enzymatic treatment on the composition, structural organization and biomechanics of meniscal tissues that are engineered by inkjetting mesenchymal stem/stromal cells (MSCs) into MEW scaffolds with aligned pore architectures that support the development of structurally anisotropic fibrocartilaginous grafts.

## 2 Materials and Methods

### 2.1 Melt Electrowriting (MEW)

One millimeter in height MEW scaffolds with a fiber diameter of 15 μm were fabricated on a custom MEW printer built in house.^39,40^ Briefly, PCL (50 000 MW, CAPA 6500D, Perstorp) was melted at a temperature of 80 and 85 °C at the barrel and around the nozzle (21G) respectively. PCL was extruded at a pressure of 0.6 bar with an initial voltage of 6 kV at a distance of 6 mm from the grounded aluminum collector plate, and a translational speed of 6 mm/s. Fibers were subsequently deposited with the predesigned architecture.

### 2.2 Isolation and Expansion of Mesenchymal Stem Cells

Bone marrow derived MSCs (bMSCs) were harvested from the femur of a 4-month old pig supplied by a local abattoir, a previously described.^41^ Briefly, the bone marrow was collected under sterile conditions from the femoral shaft, and diluted with expansion medium (XPAN), consisting of hgDMEM supplemented with 10% v/v FBS, 100 U/mL penicillin, 100 μg/mL streptomycin, and 2.5 μg/mL amphotericin B (all Gibco, Biosciences). After obtaining a homogeneous suspension using a 16G needle and filtering through a 40 μm nylon mesh, cells were counted using trypan blue containing 3% acetic acid before plating into T175 flasks for expansion. All expansion was performed at 5% pO2, and using XPAN medium supplemented with 5 ng/mL FGF2 (Peprotech), seeding at a density of 875 000 cells per T175 flask. Chondrogenic, osteogenic, and adipogenic differentiation assays were used to assess the tripotentiality of isolated MSCs. Cells were used at the end of passage 3 for all experiments.

### 2.3 Oxidized Alginate Preparation (OA)

The alginate oxidation was performed as previously described.^42^ Briefly, 1 g of alginate (MVG, Pronova Biopolymers) was dissolved in deionized water overnight at RT and mixed with sodium periodate (Honeywell) to achieve a theoretical alginate oxidation of 4% under stirring in the dark at room temperature for 24 h. The oxidized alginate was then purified by dialysis against deionized water for 3 days (MWCO 3500 Da; Fischer), sterile filtered through 0.22 μm filter, and lyophilized to obtain the final product.

### 2.4 Inkjet Bioprinting and Chondrogenic Culture

The inkjet head of the 3D Discovery multihead printing system (RegenHu) was used to deposit a cell-alginate bioink (3 × 10^7^ cells/mL in 3% (w/v) oxidized alginate). Single droplets were deposited into each well to yield 17 × 10^3^ cells/microchamber (using a valve opening time of 600 μs and a pressure of 0.1 MPa). Postprinting, a 45 mM CaCl_2_ solution in hgDMEM was added to cross-link the alginate for 5 min at room temperature. The cross-linking solution was then removed and replaced by XPAN medium. After a period of 24 h, all constructs were moved to chondrogenic differentiation medium (CDM), which consisted of hgDMEM supplemented with 100 U/mL penicillin, 100 μg/mL streptomycin (both Gibco), 100 μg/mL sodium pyruvate, 40 μg/mL l-proline, 50 μg/mL l-ascorbic acid-2-phosphate, 4.7 μg/mL linoleic acid, 1.5 mg/mL bovine serum albumin, 1× Insulin–Transferrin–Selenium (ITS), 100 nM dexamethasone, 2.5 μg/mL Amphotericin B (all from Sigma), and 10 ng/mL of human transforming growth factor-β3 (TGF-β) (Peprotech). Cells were cultured at 5% pO2 for 4 weeks with medium changes performed every 2 days.

### 2.5 Chondroitinase-ABC Treatment

On day 14 of the chondrogenic culture, constructs were treated with an enzymatic solution consisting of 2 U/mL cABC (Sigma-Aldrich and 0.05 M acetate (Trizma Base, Sigma-Aldrich) activator in hgDMEM for 4 h at 5% pO2. After the enzymatic treatment, the engineered tissues were washed with hgDMEM before the addition of CDM and the continuation of the culture period. This enzymatic treatment protocol is based on previous work in the literature.^32,34,35^

### 2.6 Live/Dead Imaging

Cell viability was assessed using the live/dead assay. Briefly, constructs were washed in PBS followed by incubation for 1 h in PBS containing 2 μM calcein acetoxymethyl (calcein AM) and 4 μM ethidium homodimer-1 (EthD-1) (both from Bioscience) for 1 h. Samples were then washed in PBS before imaging with a Leica SP8 scanning confocal microscope excited at 494 and 528 nm, and read at 517 and 617 nm.

### 2.7 Biochemical Analysis

The engineered constructs were biochemically analyzed after 4 weeks of in vitro culture. After serial washes with PBS, each construct was digested with papain (3.88 U/mL) in ultrapure water containing 0.1 M sodium acetate, 5 mM l-cysteine–hydrochloride hydrate, and 5 mM methylenediaminetetraacetic acid (EDTA) (all from Sigma). Samples were incubated in papain solution pH 6.5 at 60 °C under rotation for 18 h. Immediately after digestion, the DNA content was quantified using the Hoechst 33258 dye assay with calf thymus DNA as standard (Merck) reading at 360 nm excitation and 460 nm emission. The amount of sulfated glycosaminoglycan (sGAG) was quantified using the 1,9-dimethylmethylene blue (DMMB) dye-binding assay, with a chondroitin sulfate solution as standard (Blyscan).^43^ The 530/590 absorbance ratio was used to generate the standard curve and determine the sGAG concentration in the digested samples. Total collagen content was determined by measuring the hydroxyproline content using the dimethylaminobenzaldehyde and chloramine T assay, and a hydroxyproline/collagen ratio of 1:7.69.^44^ Briefly, samples were mixed with 100 μL of 38% HCl, and incubated at 100 °C for 18 h. After cooling, samples were centrifuge at 5000 g for 5 min, and left to dry at 50 °C for 48 h. Dried samples were then dissolved in 200 μL of ultrapure water. 2.82% (w/v) chloramine T solution was added and incubated for 20 min at room temperature in the dark, before the addition of a 50% (w/v) 4-(dimethylamino) benzaldehyde solution (both Sigma) at 60 °C for 20 min. Hydroxyproline levels were estimated from the standard curve at a wavelength of 570 nm.

### 2.8 Histological Analysis

Constructs were fixed in 4% (w/v) paraformaldehyde, dehydrated in a graded series of ethanol and xylene baths, embedded in paraffin wax, sectioned at 8 μm using a microtome (Leica Microsystems), and affixed to microscope slides. The sections were stained with hematoxylin and eosin (H&E), Alcian blue, picrosirius red, and alizarin red. For immunohistochemistry antigen retrieval was carried out by an initial treatment with Pronase (3.5 U/mL; Merck) at 37 °C for 25 min, followed by hyaluronidase (4000 units/mL; Sigma-Aldrich) at 37 °C for 25 min for collagen type I and type II. For collagen type X, the antigen retrieval method consisted of an initial treatment with Pronase (35 U/mL; Merck) at 37 °C for 5 min, followed by chondroitinase ABC (0.25 U/mL; Sigma-Aldrich) at 37 °C for 45 min. Nonspecific sites were blocked using a 10% goat serum and 1% BSA blocking buffer for 1 h at room temperature. Collagen type I (1:400; ab138492; Abcam), type II (1:400; sc52658; Santa Cruz), and type X (1:300; ab49945; Abcam) primary antibodies were incubated overnight at 4 °C, followed by 20 min treatment using a solution of 3% hydrogen peroxide (Sigma-Aldrich). The secondary antibodies for collagen type I (1:250; ab6720; Abcam), type II (1:300; B7151; Sigma-Aldrich), and type X (1:500, ab97228; Abcam) were incubated for 4 h at RT. Samples were then incubated for 45 min with VECTASTAIN Elite ABC before treating them with ImmPACT DAB EqV (both from Vector Laboratories) at RT. Slides were then imaged using an Aperio ScanScope slide scanner, while sections stained with picrosirius red were imaged using polarized light microscopy. Analysis on the fiber orientation and coherency was carried out using the OrientationJ plugin in ImageJ software.^45^

### 2.9 Scanning Electron Microscopy

For SEM imaging, samples were fixed in 3% glutaraldehyde overnight, and dehydrated in graded ethanol series before immersing the samples into hexamethyldisilazane. After drying, samples were mounted on SEM pin stubs with carbon adhesive discs and coated with gold/palladium for 60 s at a current of 40 mA using a Cressington 208HR sputter coater. Imaging was carried out in a Zeiss ULTRA plus SEM.

### 2.10 Scanning Helium Ion Microscopy

For high resolution visualization of the collagen network, samples were image with a scanning helium ion microscope (Zeiss ORION Nanofab). At the end of the culture period, samples were treated with 0.6 U/mL cABC for 24 h at 37 °C, followed by a treatment with 20.4 U/mL hyaluronidase for another 24 h at 37 °C.^46^ After the enzymatic treatment, samples were washed in PBS, and rinsed with diH2O. Finally, samples were dehydrated in a gradient of series of ethanol and dried at a critical point. Samples were then mounted and coated as previously described and imaged using a Zeiss ORION Nanofab.

### 2.11 Mechanical Testing

To investigate the mechanical properties of the engineered tissues, the different groups were tested using a single column Zwick (Zwick, Rowell) with a 10 N load cell at room temperature. Unconfined compression tests were carried out in a PBS bath as previously described.^47^ The Young’s modulus was defined as the slope of the linear phase of the resulting stress–strain curve during the ramp phase of the compression to 10% strain. The equilibrium modulus was determined as the force in equilibrium divided by the sample’s cross-sectional area divided by the applied strain, while the dynamic modulus was measured as the average force amplitude over the five cycles divided by the sample’s cross-sectional area divided by the applied strain amplitude.^48^ Uniaxial tensile tests were performed into rectangular sections as previously described.^49^ The elastic modulus was calculated from the stress–strain curves in the linear region.

### 2.12 Statistical Analysis

Statistical analysis was performed using GraphPad Prism software. Statistical differences were determined by analysis of variance (ANOVA) followed by Tukey’s multiple comparison test, or Student’s *t* test, where appropriate. Results are displayed as mean ± standard deviation. Significance was accepted at a level of *p* < 0.05. Sample size (n) is indicated within the corresponding figure legends.

## 3 Results

### 3.1 Integrating MEW and the Inkjet Bioprinting of Cell-Laden Bioinks

**Figure 1:**
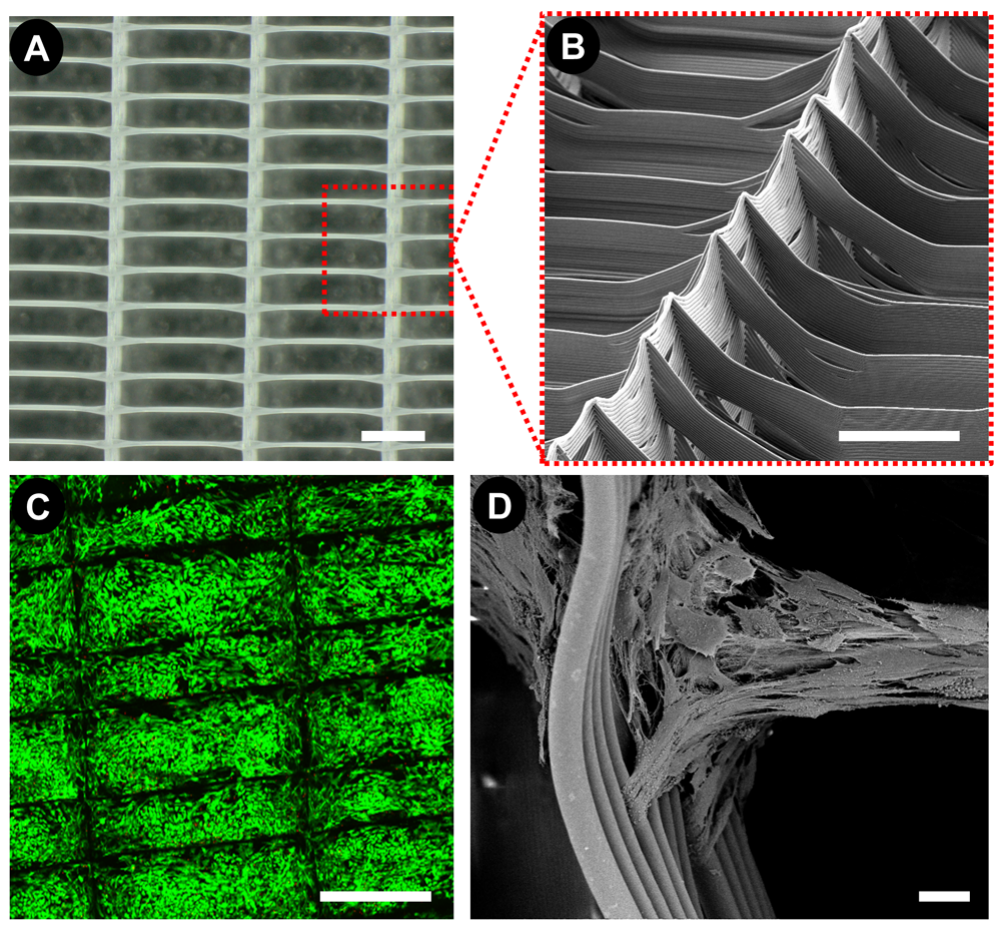
Development of the guiding microchamber system and inkjet bioprinting. (a, b) Stereomicroscope and SEM images of the MEW scaffold. Scale bars are equal to 800 μm. (c) Live/dead images (z-stacks) at day 7 after the inkjet step. Scale bar is equal to 800 μm. (d) SEM images of the cells interacting with the MEW scaffold. Scale bar is equal to 20 μm.

A previously optimized biofabrication process was used to control the deposition of MSCs into MEW scaffolds.^50^ Briefly, a MEW process was first used to produce fibrous PCL scaffolds with a pore size of 0.4 mm by 1.6 mm and a scaffold thickness of 1 mm (Figure 1A). To this end, polycaprolactone (PCL) was melted at 80 °C and deposited using a starting electric field of 6 kV and a pressure of 0.6 bar, resulting in a scaffold consisting of an array of microchambers of defined size. Scanning electron microscopy (SEM) of the scaffolds revealed the precise stacking of PCL fibers, with an average fiber diameter of 15 μm (Figure 1B). A defined number of MSCs within a supporting oxidized alginate bioink were next jetted into each scaffold pore (here termed ‘*microchambers*’) of these guiding MEW scaffolds, resulting in a relatively homogeneous distribution of cells throughout the construct.^30^ Approximately 0.56 μL of this ink was deposited into each microchamber, with each droplet containing 17,000 cells. Live/dead imaging indicated that the cells remained viable 7 days after the biofabrication process (Figure 1C). SEM analysis revealed that the cells were able to interact with and deform the PCL microfibers (Figure 1D).

### 3.2 Enzymatic Treatment Supports the Development of a More Collagenous Rich Engineered Tissue

**Figure 2:**
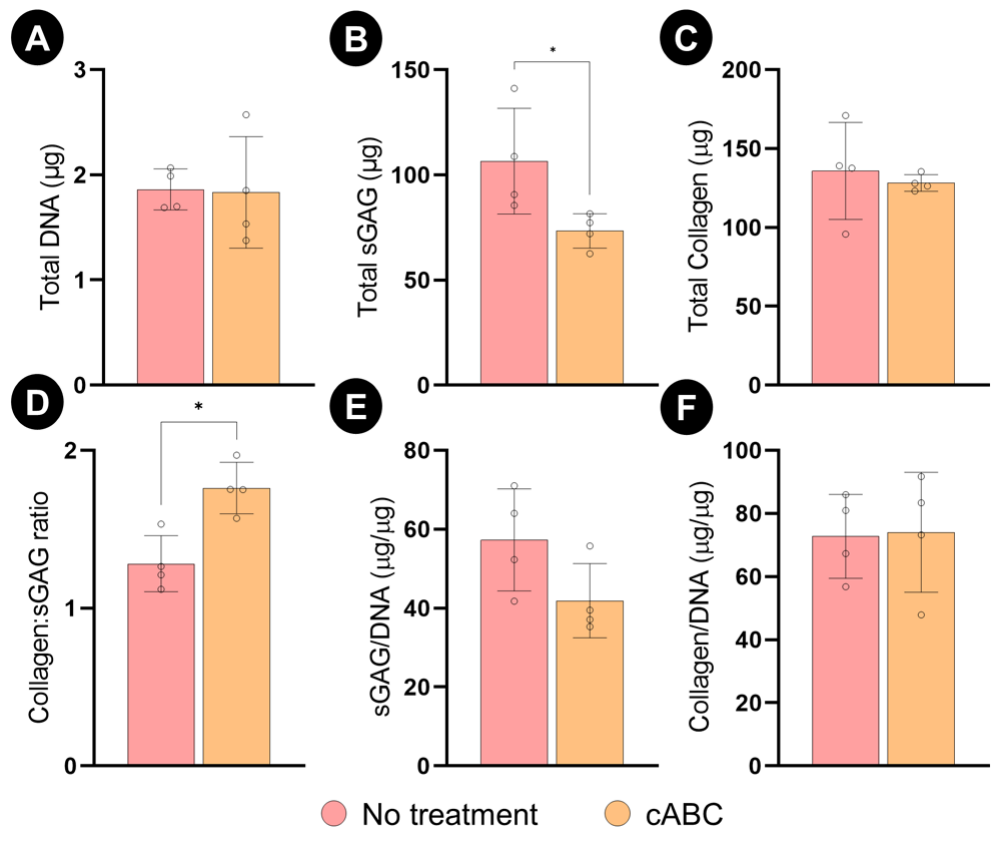
Biochemical properties of the engineered cartilage tissue following 5 weeks of in vitro culture. Quantification of (a) DNA, (b) sGAG, and (c) collagen. (d) Collagen/sGAG ratio. (e) sGAG and (f) collagen content normalized to the amount of DNA per construct. All error bars denote standard deviation, and significance was considered *p* < 0.05, *n* = 4.

We have previously demonstrated that appropriately designed MEW scaffolds can support the development of structurally organized fibrocartilage when seeded with MSCs and maintained in chondrogenic culture conditions.^50^ In an attempt to support collagen fiber maturation, here we investigated the effect of temporal enzymatic treatment on the functional development of such engineered fibrocartilaginous tissues. To this end, we exposed the engineered tissue to chondroitinase ABC (cABC) solution for 4 h on day 14 of chondrogenic culture. As expected, biochemical analysis after 5 weeks of culture indicated that cABC treatment significantly reduced total sGAG levels compared to nontreated controls (Figure 2B). cABC treatment had no effect on the total levels of DNA or collagen (Figure 2A, C). Enzymatic treatment was also found to alter the ratio of sGAG to collagen within the engineered tissue, supporting the development of a more collagen rich matrix typical of the native meniscus (Figure 2D).^1^

**Figure 3:**
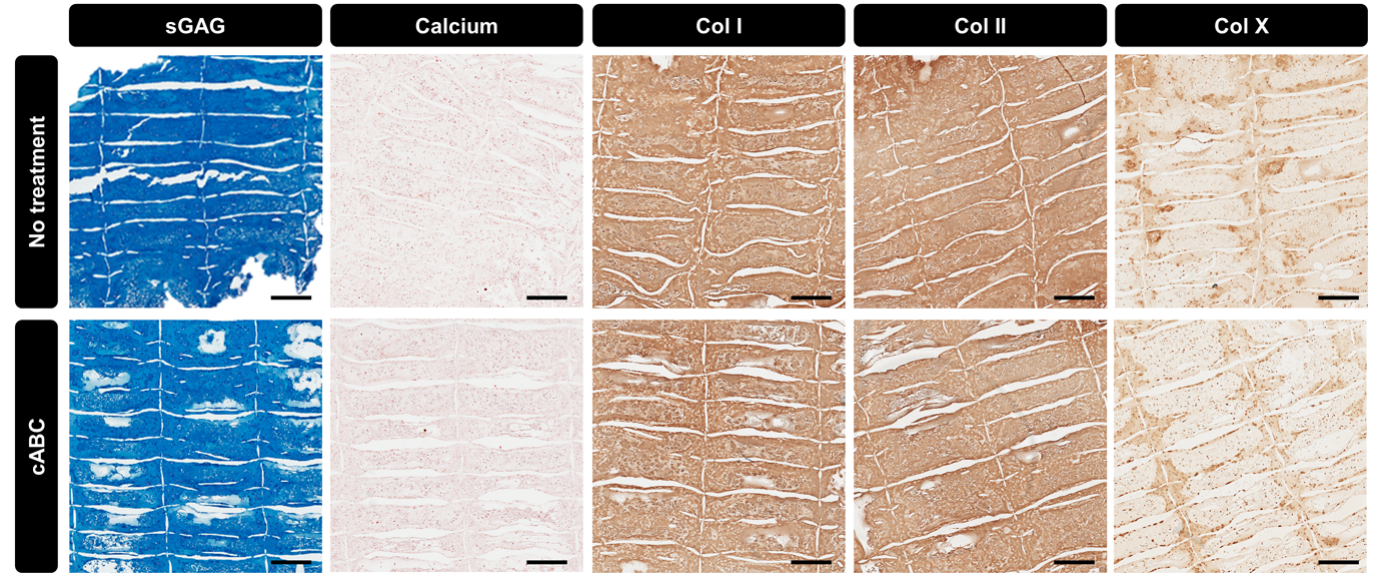
Histological analysis of the engineered cartilage tissue following 5 weeks of in vitro culture. Stained for alcian blue (sGAG), alizarin red (Calcium), collagen type I, II, and X. Scale bar is equal to 800 μm.

Having identified changes in ECM composition following the enzymatic treatment, we further investigated how cABC treatment influences tissue phenotype using histology and immunohistochemistry. As expected alcian blue staining, which stains for sGAGs, was lighter in the treatment group (Figure 3). No evidence of calcium deposition (a marker of tissue hypertrophy) was observed in either group. To further probe a fibrocartilage phenotype, immunohistochemical staining for collagen types I, II, and X were undertaken. All engineered cartilage stained intensely for collagen type I and II. Collagen type X was expressed at lower levels, with staining observed predominantly at the edges of the microchambers. This suggests that cABC treatment does not have a major impact on the tissue phenotype.

### 3.3 Enzymatic Treatment Influences Collagen Network Maturation

**Figure 4:**
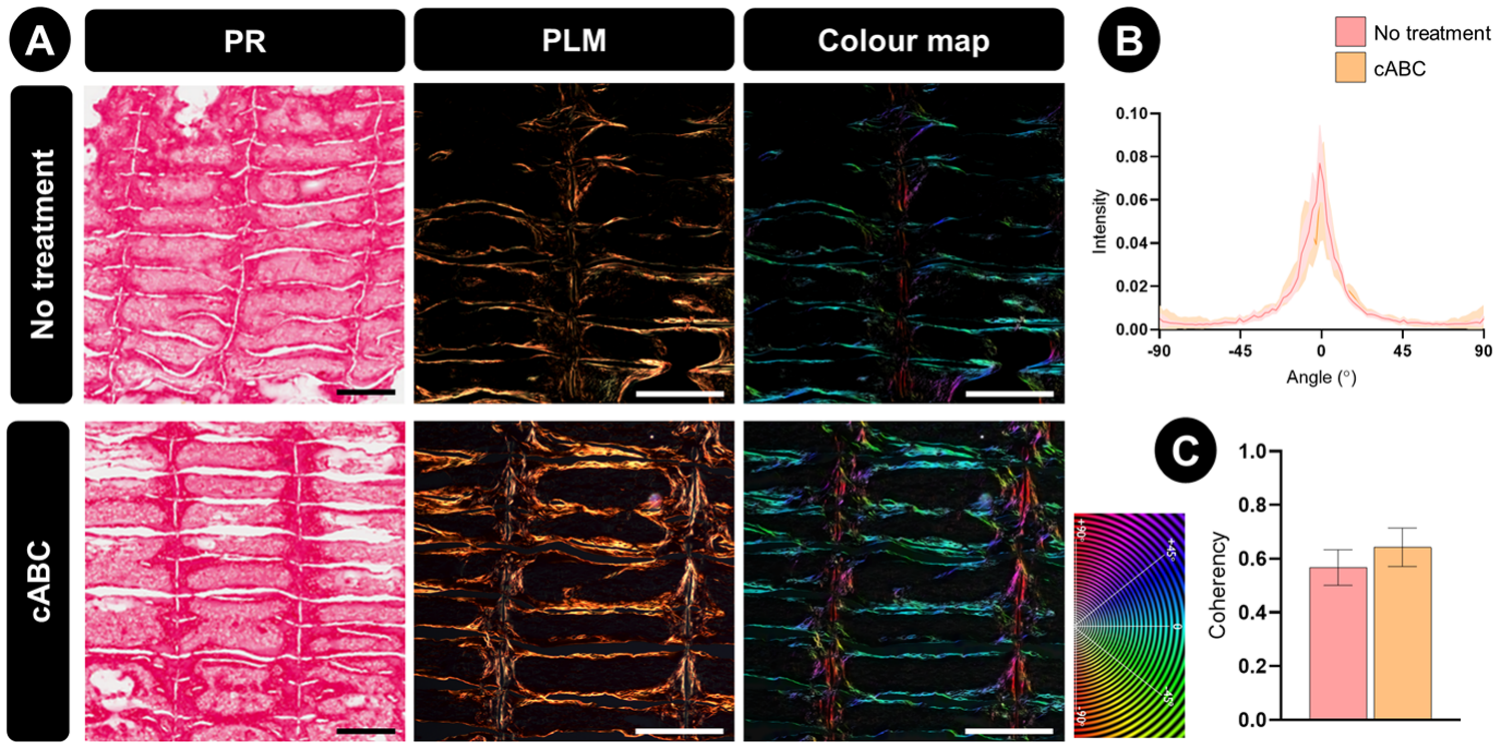
Collagen organization within the engineered tissues following 5 weeks of *in vitro* culture. (A) Picrosirius red staining, polarized light, and color map imaging of collagen fiber distributions. Scale bar is equal to 800 μm. (B) Collagen fiber directionality. (C) Fiber coherency quantification, where a value of 1 indicates fibers are aligned in the same direction, while a value of 0 indicates dispersion of fibers in all directions. All error bars denote standard deviation.

Polarized-light microscopy (PLM) was next used to investigate if enzymatic treatment influences the spatial organization of the engineered tissue. Although the intensity of picrosirius red (PR) staining for collagen deposition appeared unaffected by cABC treatment, it did result in a clear change of intensity in the color of the collagen fibers when viewed under polarized light (Figure 4A). This higher intensity is typically indicative of thickening of the collagen fibers, suggesting that cABC treatment was supportive of increased collagen fiber maturity.^51^ cABC treatment did not appear to influence collagen network alignment, with collagen fibers in the treated and untreated groups displaying clear preferential directionality parallel to the long axis of the MEW microchambers (Figure 4B). Fiber coherency, which indicates the variance of the collagen fiber distribution, was not statistically different between groups (Figure 4C). Collectively, these results suggest that cABC does not alter the fiber distribution, but that it does affect fiber maturation.

**Figure 5:**
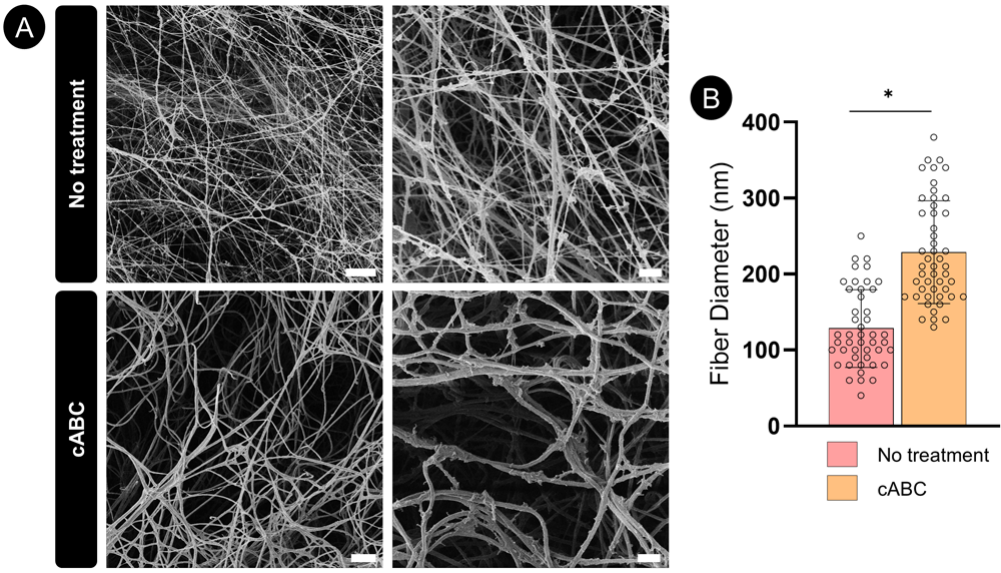
SEM analysis of the collagen organization within the engineered tissues. (A) Representative SEM images. Scale bar is equal to 5 μm on the left, and 1 μm on the images on the right. (B) Quantification of the collagen fiber diameter. All error bars denote standard deviation, significance was considered *p* < 0.05.

To further evaluate the influence of cABC treatment on collagen network development, the engineered tissues were analyzed by SEM (Figure 5A). The resulting images were quantified to determine collagen fiber diameter. Collagen fiber diameter in the nontreated groups was found to be 128 ± 52.23 nm, whereas with the cABC treatment the average fiber size increased by ∼78% to 229 ± 69.58 nm (Figure 5B). These results indicate that a one-time enzymatic treatment has a potent effect on collagen fiber maturation.

### 3.4 Enzymatic Treatment Increases the Mechanical Properties of Bioprinted Fibrocartilage Tissue

**Figure 6:**
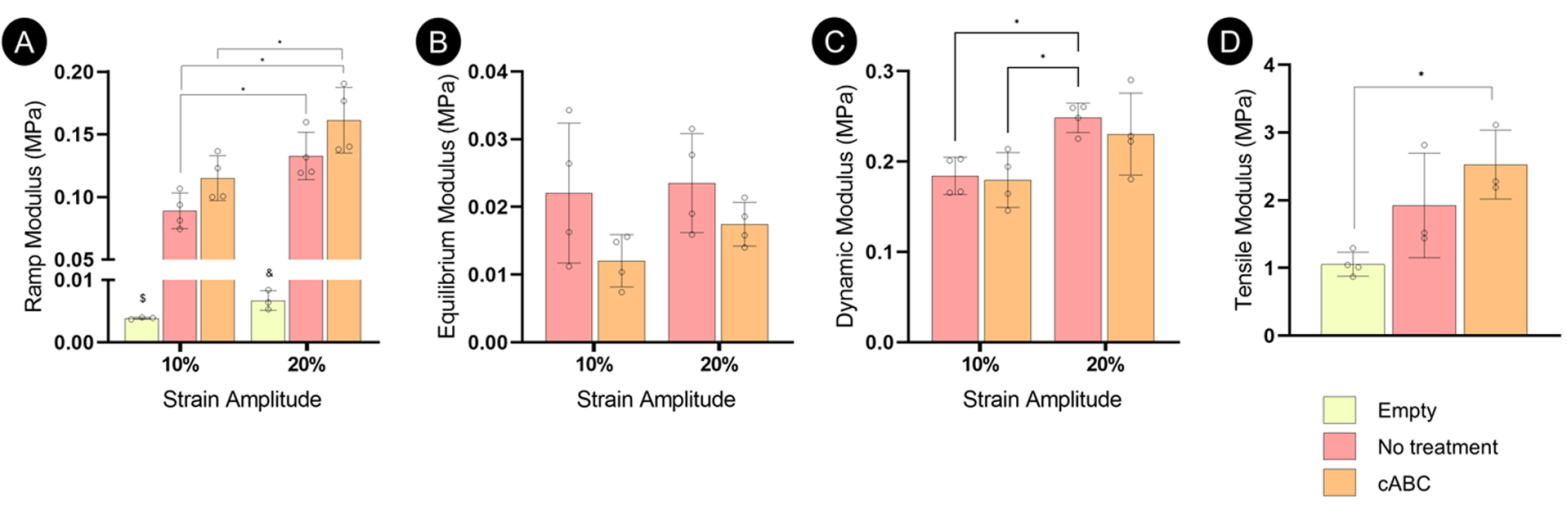
Biomechanical properties of the engineered tissues following 5 weeks of *in vitro* culture. (A) Ramp modulus. (B) Equilibrium modulus. (C) Dynamic modulus. (D) Tensile modulus. All error bars denote standard deviation, significance was considered *p* < 0.05, *n* = 4 for the compression tests and *n* = 3 for the tensile samples.

Biomechanical testing was carried out to understand the influence of cABC treatment on the functional development of the engineered tissue. No significant influence of cABC treatment was observed during unconfined compression testing, although there was a trend toward cABC treatment increasing the compressive ramp modulus of the tissue (Figure 6A). On the other hand, there was a trend toward a lower equilibrium modulus following cABC treatment, which is to be expected given the drop in sGAG content in this group (Figure 6B, C). Finally, only cABC treated tissues were found to have a tensile modulus that was significantly higher than that of an empty MEW scaffold after 5 weeks of culture (Figure 6D).

## Discussion

This study focused on investigating the effects of cABC treatment on collagen network development in bioprinted fibrocartilaginous tissue. We had previously shown that by modifying the architecture of MEW scaffolds, specifically the aspect ratio of its pores (or microchambers), that is was possible to control the directionality of collagen fibers laid down by cells jetted into such constructs.^50^ However, the tensile properties of such engineered tissues were at least an order of magnitude below native values. Here we hypothesized that a one-time enzymatic treatment would positively impact the maturation of the collagen network and hence improve the biomechanical properties of the engineered graft. We found that the temporal depletion of sGAGs within the engineered tissue following cABC treatment was associated with an increase in collagen fiber diameter without any detrimental effect on the tissue phenotype or collagen fiber directionality. Furthermore, this increase in collagen fiber thickness correlated with superior biomechanical properties.

MEW allows for the fabrication of well-defined polymeric scaffolds with specific geometries.^39,52^ Due to residual charges around the deposited fibers, it can be challenging to produce large scale scaffolds using this additive manufacturing approach. However, iteratively increasing the electric field strength during the manufacturing process can overcome such challenges.^39,53,54^ In this work, we fabricated 1 mm thick scaffolds with a fiber thickness and a pore architecture that has previously been shown to direct collagen fiber alignment parallel to the long axis of the scaffold pore walls.^50^ After jetting MSCs into the MEW scaffolds, we observed a homogeneous distribution of cells within the pores of the scaffold. Our work indicates that this convergence of biofabrication strategies (MEW and inkjetting) is a potent strategy for the spatial patterning of cells within large MEW scaffolds with user-defined architectures, potentially enabling the engineering of spatially complex tissues.

Although this multiple tool biofabrication strategy enables the engineering of spatially organized tissues at a millimeter scale, it is still challenging to produce constructs with collagen content and mechanical properties comparable to native tissues. Here, we demonstrate that the use of cABC as a remodeling enzyme reduces the total sGAG content within the engineered tissue, thereby modulating the ratio of collagen to sGAG without compromising cell viability. Since the native meniscus is a predominantly collagen rich tissue, such treatments support the engineering of tissues that better recapitulate the biochemical content of the native meniscus.^55^ In line with previous work, we observed that the enzymatic treatment did not negatively impact subsequent ECM synthesis.^33,37,56^ Furthermore, enzymatic treatment had no effect on the resulting tissue phenotype, specifically the types of collagen deposited within the MEW scaffolds. No evidence of calcium deposition, a marker of hypertrophy and progression along an endochondral pathway, was observed in either group. Therefore, the use of cABC during chondrogenic differentiation represents a potent approach to modulate the biochemical composition of the engineered tissues without any detrimental effect on cell phenotype.

Temporal enzymatic treatment was associated with the development of larger diameter collagen fibers within the engineered tissue, as evident by polarized light microscopy and SEM. The presence of proteoglycans is known to impact collagen fibril morphology and formation kinetics by hindering lateral growth during fibril formation, leading to suggestions that their removal by cABC treatment can enhance collagen network development in engineered tissues,^37^,^57^.^58^ Furthermore, it has been shown that increasing sGAG content is inversely correlated with decreasing collagen fibril size, indicating that sGAGs are important regulators of collagen network formation.^59^ Importantly for the engineering of structurally organized fibrocartilage tissue, such changes in collagen fibril diameter did not have any detrimental effect on collagen alignment within the MEW scaffolds. This meant that cABC treatment not only enhanced collagen network maturity, but also increased the tensile properties of the graft. In spite of these improvements, the tensile properties of the bioprinted meniscal grafts were still substantially lower than that of the native tissue. In the healthy meniscus, the collagen fibrils are organized together in bundles approximately 20–200 μm wide.^4^ Therefore, while temporal enzymatic treatment represents a promising strategy to promote collagen network maturation, additional biochemical and biophysical cues are likely required to promote further tissue development *in vitro*. It is also important to recognize the importance that proteoglycans play in the biomechanics of hyaline cartilage and fibrocartilaginous tissues such as the meniscus. For example, they contribute to the tissue’s high-water content and enable the resistance to large compressive forces generated within the knee.^1^ Therefore, the ideal tissue engineering strategy would allow both collagen fiber maturation and the eventual accumulation of sGAG, in relative ratios and organizations that are mimetic of the native tissue.

In conclusion, this work describes a biofabrication strategy to direct collagen fiber alignment and maturation in bioprinted fibrocartilage tissue, where temporal depletion of sGAGs through enzymatic treatment with cABC facilitates the assembly of larger diameter collagen fibers. Chondrogenically primed MSCs produce high levels of sGAG in culture, which appears to impact collagen fiber formation and the functional development of the engineered tissue. The application of cABC to sGAGs in the early stages of tissue development supports an increase in collagen fiber size and graft mechanical properties without any detrimental effect on cell viability or tissue phenotype. These findings support the inclusion of enzymatic treatments when bioprinting fibrocartilaginous tissues as a means of generating more functional and biomimetic grafts.
